# Assessment of Incorporation of Lessons From Tobacco Control in City and County Laws Regulating Legal Marijuana in California

**DOI:** 10.1001/jamanetworkopen.2020.8393

**Published:** 2020-06-19

**Authors:** Lynn D. Silver, Amanda Z. Naprawa, Alisa A. Padon

**Affiliations:** 1 Public Health Institute, Oakland, California

## Abstract

**Importance:**

As marijuana continues to be legalized in many states, little is known about best regulatory practice, exposing the population to significant potential harm.

**Objective:**

To assess the extent to which potential best practices, including those from tobacco control, were incorporated into state and local marijuana policies in California.

**Design and Setting:**

California legalized medical marijuana in 1996 and adult recreational use in 2016, effective in January 2018. A cross-sectional study with data collection and analysis from February 1 to November 30, 2019, measured the adoption of potential demand reduction and youth protection best practices, including restrictions on sales, products, marketing, warnings, and taxation. Laws in effect by January 31, 2019, were verified and all 539 California local jurisdictions were studied.

**Main Outcomes and Measures:**

Adoption of potential best practices in marijuana laws for demand reduction and youth protection.

**Results:**

The laws of 534 of California’s 539 jurisdictions (99%) were successfully identified; 263 of these 534 jurisdictions (49%) allowed any retail sale of marijuana, covering 57% of the state’s population. More than one-third of jurisdictions allow sales of marijuana for adult recreational use (203 of 534 [38%]); of those, 122 allow storefront dispensaries and 81 allow sales by delivery only. A total of 257 of 534 jurisdictions (48%) allow medical sales. Of 147 jurisdictions allowing medical or adult use dispensaries, 93 (63%) limited the number of licenses, with a mean of 1 store for every 19 058 residents (range, 154-355 143). The state imposed no limits on number of dispensaries or deliverers. Forty-two jurisdictions increased the state-specified distances required between dispensaries and schools. Only 8 jurisdictions allowing retail sales imposed restrictions on products exceeding state regulations; 1 prohibited sale of flavored products, 3 prohibited sale of marijuana-infused beverages, and 5 imposed additional restrictions on edible marijuana products. No jurisdictions limited potency of products sold, although 1 established a potency-linked tax. The state did not limit or tax potency, except for establishing a standard 10-mg dose of tetrahydrocannabinol for edible marijuana products, nor did they limit manufacturing or sale of flavored products. The state required only a health warning in 6-point font on packages. Twenty-seven jurisdictions required additional health warnings in stores or on packages, 27 allowed onsite consumption of marijuana products, and 13 allowed marijuana-related events. More than half of jurisdictions legalizing any cannabis commerce (154 of 289 [53%]) did not tax marijuana locally and little revenue was captured for prevention. Much of the state excise and cultivation taxes is slated for youth substance use prevention and treatment.

**Conclusions and Relevance:**

In implementing legalization of marijuana in California, local policies varied widely. Where marijuana was legalized, many lessons from tobacco control to reduce demand, limit harm, and prevent youth use were not adopted, potentially creating greater risk of harm.

## Key Points

**Question:**

What legal limits have cities and counties adopted since California legalized marijuana use for adults, and to what extent do these legal limits incorporate public health recommendations and lessons from tobacco control?

**Findings:**

This cross-sectional study of all 539 California cities and counties found that jurisdictions differed widely in how they regulated marijuana and that key public health recommendations and lessons from tobacco control have generally not been adopted, although local innovation is emerging.

**Meaning:**

Jurisdictions undergoing marijuana legalization may need to consider incorporating policy lessons from control of other legal but harmful products to reduce potential future burden of harm.

## Teaser

This cross-sectional study assesses the extent to which potential best practices, including those from tobacco control, were incorporated into state and local marijuana policy in California.

## Introduction

Marijuana continues to be legalized in many states, generally with limited public health input. Although valid medicinal applications exist, the National Academies of Science, Engineering, and Medicine concluded that substantial evidence suggests that marijuana use is also associated with significant harms, including psychosis, schizophrenia, problem marijuana use, motor vehicle collisions, low birth weight, and respiratory symptoms.^1^ Evidence is emerging regarding the association of marijuana use with youths’ cognition^2,3^ and cardiovascular disease,^4,5,6^ as well as other areas, and the 2019 vaping epidemic demonstrated the hazards of rapid product innovation without due evaluation of safety.^7^ With widespread lifetime and adolescent use of marijuana, reaching 43.6% of 12th-grade students nationally,^8^ and 51.5% of 18- to 25-year-olds in 2018,^9^ even modest increases in risk may have a significant effect on population health. Vaping of marijuana in the past 30 days, which typically involves high-potency concentrates, increased from 5% of 12th-grade students in 2017 to 14% in 2019, with 3.5% vaping near daily in 2019.^10^ The potential magnitude of mental health effects associated with the growing market of high-potency marijuana products is evidenced by estimates of the population-attributable fraction of first-episode psychosis due to use of high-potency marijuana (>10% tetrahydrocannabinol [THC]) at 12% (range, 1.9%-50.3%) in 11 primarily European cities studied, and by elevated risk for first-episode psychosis found in individuals using these products daily.^11^ Treatment data also suggest reason for concern. In 2014, marijuana was the leading drug used by clients entering drug treatment in a study of 22 European countries, representing 46% of all new clients, up from 29% in 2003.^12^ Both marijuana-related new clients and daily users in treatment more than doubled between 2003 and 2014.

Prior to legalization of adult use of marijuana in California, as legalization advanced nationally, identification of key policy concerns and calls for caution emerged. Barry and Glantz recommended that “to protect public health, marijuana should be treated like tobacco, legal but subject to a robust demand reduction program modeled on evidence-based tobacco control programs before a large industry (akin to tobacco) develops and takes control of the market and regulatory environment.”^13^^(p2)^ Authors noted that the transition from small-scale marijuana growers and retailers to large-scale industrial consolidation and marketing would bring risks, including aggressive lobbying, campaign contributions, and efforts to create favorable regulation. Richter and Levy^14^ noted the parallels between modern trends in marijuana product diversification and past transformations of tobacco to a deadly industrialized product designed to boost nicotine delivery and enhance addictive potential and palatability. Volkow et al at the National Institutes of Health raised concerns over the potential effects of rising product potency and of use on the developing brain.^15^ Subsequently, in 2019, Ayers et al^16^ called attention to emerging patterns of marijuana branding, marketing health claims, lack of health warnings, and appeals to youths and called for federal regulation. California’s tobacco control oversight experts called for broad application of lessons learned from tobacco control to commercial marijuana.^17^ Others called for legalization processes to intentionally advance social equity through criminal justice policy, offering economic opportunity to communities hard hit by the war on drugs, and reinvesting revenues in those communities.^18^

In November 2016, a California ballot initiative, Proposition 64,^19^ successfully legalized production and sale of marijuana for adult use, 20 years after legalization of medicinal use of marijuana in the state. An important part of that initiative was the assurance that local control would be preserved and cities and counties would have broad discretion to allow legal marijuana commerce, or not, and to regulate its practice. Decriminalization of possession of allowable quantities of marijuana was assured statewide. In 2017 and 2018, the state created a regulatory framework for legal cultivation, manufacturing, and retailing of marijuana, which generally prioritized facilitating the shift from the illegal market to the legal market rather than demand reduction strategies.

The first legal marijuana dispensaries for adult use in California opened January 2018. Three marijuana industry behaviors—extensive increases in potency (percentage of THC in products), manufacturing of products to attract youths, and aggressive marketing—that were directly adopted from tobacco industry practices became immediately evident across the state. Despite the threat to public health, state regulations failed to constrain these practices, even though California has led tobacco control efforts in the United States and pioneered tobacco control policies such as public smoking bans, flavored product bans, and electronic cigarette bans.

Cities and counties are often “laboratories” of innovation in public policy, and, notably, in tobacco control. Because California law allowed significant local control, we therefore asked: To what extent have recommendations from the public health community and potential lessons from tobacco control and other legal, but harmful, products been adopted in the marijuana legalization process?

## Methods

In a cross-sectional study with data collection and analysis from February 1 to November 30, 2019, we studied laws and regulations in California to understand the extent to which public health recommendations and tobacco control best practices, and in some cases, alcohol control best practices with evidence of effectiveness and potential relevance, had been incorporated into marijuana legislation by January 31, 2019. We followed the Strengthening the Reporting of Observational Studies in Epidemiology (STROBE) reporting guideline. The jurisdiction law review reported here was determined not to be human participant research by the Public Health Institute Institutional Review Board.

### Identification of Potential Best Practices

Selected practices were identified during an earlier literature review and national consultation with 62 stakeholders during key informant interviews conducted from 2017 to 2019 with experts in marijuana, tobacco, and alcohol regulation, the First Amendment, tobacco and alcohol law, local government, community organizing, criminal justice, and substance abuse and marijuana research, as well as marijuana industry participants. Collectively, the resuts of these interviews informed production of 2017 model ordinances for marijuana retailing, marketing, and taxation for California local government.^20,21^ Potential best practices identified included restrictions on retail outlets, buffer zones, certain product types, delivery, marketing, and conflicts of interest, as well as requiring preservation of smoke-free air, health warnings, pricing and taxation measures, and equity policies in licensing, hiring, and revenue capture.^17,18,22,23,24,25,26,27^ Although only practices considered legally defensible were recommended, descriptions of local measures were collected regardless of whether they went beyond recommendations (eg, bans on television and radio advertising).

### Verification of Laws

Local laws of 539 California cities and counties were verified using the CannaRegs commercial database, complemented by verification on jurisdictions’ websites and the Municode database of municipal law. When status remained unclear, city or county clerks were contacted directly. Because San Francisco is both a city and a county, it was counted only as a county, leaving a denominator of 539 jurisdictions: 58 counties and 481 cities. Laws passed through January 31, 2019, were included. State law and regulation was verified through the state cannabis portal.^28^ Random samples of cities (5%) and counties (10%) were iteratively coded by 2 independent coders (A.Z.N. and A.A.P.) and tested for interrater reliability, which reached 97% agreement. Remaining jurisdictions were coded by a single public health lawyer (A.Z.N.). Laws were coded as affirmatively allowed, affirmatively prohibited, or silent, which meant state law would apply, except in the cases of onsite consumption and temporary events that require affirmative local allowance.

### Statistical Analysis

Independent-samples *t* tests were conducted to compare differences in legalization between cities and counties, and by population size, assuming unequal variances. All *P* values were from 2-sided tests and results were deemed statistically significant at *P* < .05. Analyses were performed using Stata, version 15.1 (Stata Corp).^29^

## Results

### Retail Sales

The marijuana laws of 534 (99%) of California’s 539 cities and counties were successfully identified. Of these 534 jurisdictions, 263 (49%) allowed any retail sale of marijuana, either medical and/or adult use, covering 57% of the state’s population. More than one-third of jurisdictions (203 of 534 [38%]) allowed adult-use sales of marijuana; of those, 122 allowed storefront dispensaries and 81 allowed sales by delivery only. Nearly half of jurisdictions (257 of 534 [48%]) allowed medical sales (Table 1). The state allows both medical and adult-use sales unless locally prohibited. Cities were significantly less likely than counties, whose laws apply only to their unincorporated areas in California, to have legalized any marijuana retail (227 of 476 [48%] vs 36 of 58 [62%]; *P* = .04) and were significantly less likely to have legalized any adult-use retail (172 of 476 [36%] vs 31 of 58 [53%]; *P* = .01). Jurisdictions that had legalized any marijuana retail activities were more populous (mean [SD], 85 740 [283 718]) compared with jurisdictions that had not legalized marijuana retail activities (mean [SD], 63 782 [91 798]), but this difference was not significant (*P* = .23).

**Table 1.** Commercial Marijuana Activities Allowed in California Cities and Counties as of January 2019^a^

Activity type	No. (%)			
	Medical and adult use allowed	Adult use banned, medical use only allowed	Medical use banned, adult use only allowed	Neither medical nor adult use allowed
**Cities (n = 476)**				
Retail				
Storefront	97 (20)	22 (5)	7 (2)	350 (74)
Delivery	155 (33)	55 (12)	3 (1)	263 (55)
Any form	166 (35)	55 (12)	6 (1)	249 (52)
Cultivation	125 (26)	14 (3)	3 (1)	335 (70)
Manufacturing	135 (28)	17 (4)	1 (0.2)	324 (68)
**Counties (unincorporated area) (n = 58)**				
Retail				
Storefront	18 (31)	3 (5)	0	37 (64)
Delivery	30 (52)	5 (9)	0	23 (40)
Any form	31 (54)	5 (9)	0	22 (38)
Cultivation	22 (38)	3 (5)	2 (4)	31 (54)
Manufacturing	23 (40)	0	1 (2)	34 (59)
**All cities and counties (n = 534)**				
Retail				
Storefront	115 (22)	25 (5)	7 (1)	387 (73)
Delivery	185 (35)	60 (11)	3 (1)	286 (54)
Any form	197 (37)	60 (11)	6 (1)	271 (51)
Cultivation	146 (27)	17 (3)	5 (1)	366 (69)
Manufacturing	157 (29)	17 (3)	2 (0.4)	358 (67)

^a^ Data include 58 counties and 476 cities; marijuana-related regulatory data could not be found for 5 of California’s 481 cities. If a jurisdiction is silent then state law applies. The City and County of San Francisco were treated as a county. Although 51% of jurisdictions ban all retail activity, state regulation under judicial review allows delivery anywhere in the state.

### Location and Density of Retail Establishments

Of 147 jurisdictions allowing medical or adult use storefront commerce, 93 (63%) limited the number of dispensaries, with a mean of 1 store for every 19 058 residents (range, 154-355 143) (Table 2). The state imposed no limits on the number of dispensaries or delivery businesses that could be licensed. Forty-two jurisdictions imposed a buffer between retailers and schools greater than the state-required 600 feet, yet 6 jurisdictions allowed retailers to locate closer to schools than the state’s requirement, at a mean of 258 feet. More than 100 jurisdictions added establishments to the state’s list of “sensitive use” sites from which storefront dispensaries must be distanced, which consisted of kindergarten through grade 12 schools, day care centers, or youth centers. Locally adopted examples included colleges, public beaches, libraries, tutoring centers, and recreation centers. More than one-third of jurisdictions imposed buffers between retail locations, with a median of 600 feet.

**Table 2.** Adoption of Potential Demand Reduction Best Practices in Marijuana Regulation in California Jurisdictions Allowing Any Retail Sale of Medical or Adult-Use Products as of January 2019

Regulatory practice^a^	No./total No. (%)^b^
Cap on dispensaries	93/147 (63)
Ratio of dispensaries to population, mean (range)	1:19 058 (1:154-1:355 143)
Buffers from schools >600 ft	42/147 (28)
Prohibited onsite consumption	
Banned	79/147 (54)
Silent	41/147 (28)
Temporary events prohibited	17/263 (7)
Restrictions on products	8/263 (3)
Attractive to youth	5/263 (2)
Flavors	1/263 (0.4)
Beverages	3/263 (1)
Potency	0
Edible products	5/263 (2)
Price discounts prohibited	5/263 (2)
Minimum price required	0
Any tax on retail	120/263 (46)
Size of retail tax gross receipts averaging adult use and medicinal, median (range), %	5 (0-15)
Any advertising restrictions	74/263 (29)
Any restrictions on health claims	1/263 (0.4)
Additional health warnings required	27/263 (10)

^a^ These provisions refer to measures that go beyond state law.

^b^ Denominator for cap on dispensaries, onsite consumption, and buffers consists of jurisdictions that allow any storefront retail sale (n = 147); denominator for all other variables consists of jurisdictions that allow any storefront and/or delivery retail sales (n = 263).

### Equity in Licensing and Criminal Justice

Provisions to promote economic equity and diversity in marijuana licensing were limited to 5 of the largest cities. Oakland, Long Beach, and the city of Los Angeles gave a defined class of “equity” applicants priority in licensing and a reduction in certain costs, and required that certain percentages of employees be low-income, local, or transitional workers. Sacramento also had equity licensing priority and reduced costs, and San Francisco had equity licensing priority and employee requirements. The state did not establish an equity licensing system. Proposition 64 established the right to expunge certain past marijuana convictions, and state legislation subsequently approved a process for automatic expungement, reducing barriers for eligible individuals to benefit.^30^

### Restrictions on Allowable Marijuana Product Types for Sale

Among jurisdictions allowing retail sale, only 8 imposed restrictions on types of marijuana products for sale, beyond state regulations. One jurisdiction, Contra Costa County, pioneered the prohibition of sale of flavored products for combustion or inhalation, 3 jurisdictions (Pasadena, Mono County, and Chula Vista) prohibited the sale of marijuana-infused beverages resembling “alcopops”^31^ (such as marijuana orange soda or iced tea), 5 jurisdictions restricted products appealing to youths, and 5 jurisdictions imposed restrictions on edible marijuana products beyond state regulations. No jurisdictions limited the potency of products sold, although 1 jurisdiction established a potency-linked tax. The state did not limit or tax potency, except for establishing a maximum 10-mg THC dose for edible marijuana products (100 mg total per package), nor did they limit manufacturing or sale of flavored products, such as flavored vaping liquids or prerolled cigarettes, although state regulations did create restrictions on products resembling existing foods or with characteristics that were particularly attractive to children.

### Health Warnings and Claims

Although the state required only a limited health warning in hard-to-read 6-point font on packages, whose text was defined in the ballot initiative, 23 jurisdictions required additional health warnings in stores and 4 jurisdictions required additional health warnings on packages. No jurisdiction required warnings on advertising. Although the state prohibited only misleading or unsubstantiated health claims, 1 county, Mono, prohibited all health-related claims on marijuana labels, any advertising or marketing, and in retailer names.

### Restrictions on Advertising

Seventy-four jurisdictions limited advertising in some way, primarily through limited business signage. Fourteen jurisdictions prohibited billboards and other outdoor advertising. Five jurisdictions limited advertising on television, on the radio, online, or in print, and 5 jurisdictions prohibited advertisements attractive to youths more explicitly than the state’s prohibition. The state did not require warnings on advertisements and used regulation to weaken Proposition 64’s prohibition on billboards on state and interstate highways that cross state borders, limiting its application to roads within 15 miles of the state border.^32^ State law does require that advertisements be 1000 feet from schools, daycare centers, playgrounds, or youth centers, and that advertising and marketing not be designed to appeal to underage consumers.

### Smoke-Free Air and Onsite Consumption

Twenty-seven jurisdictions allowed on-site consumption of marijuana in some form at retail locations, all of which allowed either smoking (24), vaping (27), or both (24) on the premises, 3 of which allow use by staff only. Thirteen jurisdictions explicitly established a permit system for marijuana-related temporary events, while 21 jurisdictions banned them and most jurisdictions were silent. The state allows both on-site consumption and marijuana-related temporary events if locally permitted. Although California laws prohibit smoking marijuana in most workplaces or in any place where smoking cigarettes is prohibited by law, these local exceptions are now in effect.

### Price and Taxation Measures

Of 289 jurisdictions legalizing any commercial marijuana activity (including retail, cultivation, or manufacturing), 154 (53%) did not tax marijuana activity locally; 119 (41%) passed a “general” tax, which in California is a tax that the governing authority can use for any purpose; 7 (2%) passed a general tax with an advisory committee guiding revenue use; 3 (1%) passed a tax that earmarked revenue, dedicated in different cases to police and law enforcement, fire services, parks and recreation, repairing city streets, or enhancing community centers; and 6 (2%) passed “fees.” Cathedral City taxed the highest-potency marijuana concentrates, such as “shatter” (a brittle, glass-like high-potency concentrate), at 8 times the price of lower-potency products. Little local revenue was captured for prevention or reinvestment in low-income communities. Only 5 jurisdictions prohibited discounting, such as redemption of coupons, discount days, or other promotions, and none implemented a minimum price law, all of which are price policies that have been used in tobacco control. The state levied a 15% excise tax on retail sales in addition to a cultivation tax, much of which is slated for investment in prevention of substance use by youths and in communities but did not constrain discounting other than prohibiting distribution of free products, nor did it create a floor price.

### Conflicts of Interest

Fifty-three jurisdictions added some form of prescriber conflict of interest rule, such as no marijuana prescribers may work as staff or be owners or employees in retail outlets. The state prohibited those involved in marijuana regulation, enforcement, or appeals from holding marijuana licenses or financial interest, and persons licensed for testing laboratories may not hold other marijuana licenses. Neither state nor local government prohibited those with marijuana financial interests from participation in advisory bodies, and such participation is occurring.

## Discussion

Our review reveals important gaps in the regulatory scheme for marijuana in California cities and counties. Many fundamental lessons from tobacco control to reduce demand, limit harm, and prevent marijuana use by youths have gone largely ignored, leaving state law setting the standard. Nevertheless, in communities that have opted to legalize marijuana, examples are emerging of local policy innovation for reducing demand and protecting youths. Limits on retail outlets are the most common. The first prohibition on flavored products was passed in 2018, as was the first ban on vaped marijuana later in 2019. However, limitations of high-potency or flavored marijuana product types, industry practices associated with risk of addiction and psychosis, and risk of youth initiation have received little local attention. Most state residents are exposed to aggressive marketing practices such as prominent billboards promoting marijuana use. They are not informed by clear and salient health warnings such as those used on marijuana products in Canada or tobacco products in the United States. Local onsite consumption permits have been associated with smoke-filled lounges and outdoor marijuana events, such as legal sales at concerts, fairs, or park events, which may threaten decades of progress in smoke-free workplaces and outdoor air.

State laws and regulations neglected to limit retail outlets. State, like local, provisions on marketing and advertising are relatively weak, even when taking into account protections on commercial speech. The state does tax and invests some tax revenue in prevention of substance use and other community-based investments. State law and regulation does not restrict manufacturing or sale of flavored products—a well-recognized industry strategy to attract youths—despite promoting a large-scale “Flavors Hook Kids” campaign for tobacco products in the same time period.^33^ It allowed products of any potency, even those with more than 90% THC, as well as marijuana-infused sodas mimicking “alcopops” and a wide range of edible marijuana products. The entire legal marijuana market is being permitted by state and local regulators to shift to high-potency flower and concentrates in California and elsewhere.^34,35^ Similar manipulation of nicotine content to increase addiction was a tobacco industry strategy condemned in the landmark 2006 decision *US v Philip Morris*.^36^ This strategy has permitted, for example, even products such as a grape-flavored vaping cartridge in a hot pink memory stick–like device, with the equivalent of 78 unmetered “standard” 5-mg THC doses^37^ in 1/50th of 1 ounce (78% THC or 392 mg) to be sold legally. These, like flavored electronic cigarettes, may increase the risk of addiction in youths.

Many California communities reacted to legalization of marijuana by delaying or rejecting local commercial activity. The state then partially overrode voter-approved Proposition 64 guarantees of local control, promulgating regulation allowing any delivery licensee to deliver marijuana products anywhere in the state.^38^ This measure was challenged by local government and continues in the courts. These conflicts may reflect disparate visions for legalization: one prioritizing industry growth, revenue, and elimination of illicit sales; a second rejecting legalization or wishing it to occur elsewhere; and a third allowing legal commerce but prioritizing public health and demand reduction.

### Strengths and Limitations

This study has certain strengths, including the near-complete coverage of California jurisdictions, as well as providing the first snapshot of California local law. Findings are also consistent with recent work on local policy in Washington state.^39^

Nevertheless, limitations should also be noted. This study describes local regulations 1 year after adult-use sales of marijuana began. Regulation continues to evolve, and we will assess change annually. Second, we examined only local marijuana laws. Other local laws addressing issues such as zoning, advertising, or smoke-free air may include relevant provisions such as global bans on billboards that were not captured. Frameworks for legalization and local control vary widely between states, and these findings cannot be generalized. However, the concerns identified and potential best practices may be broadly relevant for national, state, and local marijuana regulation, even where local authority for adoption is absent. Importantly, the fundamental questions of whether legalization leads to net public health benefit or harm and whether these “best practices” work remain unanswered. These early descriptive data provide a valuable basis for future research on health and social outcomes in association with variations in the rigor or laxity of local policy after state legalization.

## Conclusions

In California, the nation’s largest state, policy lessons from tobacco control and other legal, but harmful, products went largely unheeded by cities and counties. Policy evaluation research is needed to understand the effects on health of regulatory paths taken. As the legal marijuana industry consolidates and its power expands, communities may wish to consider precautionary incorporation of these policy lessons into marijuana regulation from the start, potentially reducing risk of failing in our responsibility to protect youths and health for decades to come.^13^
